# Evaluation of bioactive extract nanoparticles on pulp stem cell behavior relevant to dental care using chemical composition of gelatin-Arabian gum nano polymer

**DOI:** 10.22038/IJBMS.2024.76467.16548

**Published:** 2024

**Authors:** Xiaoni Tan, Moli Zhang, BiBo Tu

**Affiliations:** 1 Department of Endodontics, Changsha Stomatological Hospital, No.389, Youyi Road, Tianxin District, Changsha 410008, Hunan Province, China

**Keywords:** Bioactive plant extracts, Chemical stability, Gelatin polymer scaffold, Migration, Proliferation, Pulp stem cells

## Abstract

**Objective(s):**

This study aimed to investigate the impact of bioactive plant extracts on the proliferation and migration of dental pulp stem cells (DPSCs) and their potential implications for dental care, focusing on the nurse-caring aspect.

**Materials and Methods:**

TDPSCs were cultured on gelatin polymer scaffolds mimicking the extracellular matrix (ECM) environment. Bioactive plant extracts with antibacterial, anti-inflammatory, and anti-oxidant properties were incorporated into the gelatin polymer at concentrations ranging from 0.1% to 2.0%. Proliferation and migration assays were performed, considering nurse-caring practices during the experiments.

**Results:**

Treatment with specific bioactive plant extracts significantly enhanced DPSC proliferation, showing a 2.5-fold increase compared to the control groups. The migration assay revealed a substantial increase in cell migration distance, with treated cells covering an average distance of 400-500 μm compared to 220-260 μm in the control group. Treated cells also exhibited improved viability and metabolic activity, with a 30% increase in cell viability and a 10-20% increase in metabolic activity compared to the control group.

**Conclusion:**

This study demonstrates that bioactive plant extracts have the potential to enhance DPSC proliferation, migration, viability, and metabolic activity. These findings support the use of these extracts in dental care, benefiting from the nurse-caring practices.

## Introduction

In recent years, there has been a growing interest in utilizing natural compounds and stem cells for innovative approaches in dental care. Among these approaches, bioactive plant extracts and pulp stem cells have emerged as promising therapeutic agents due to their regenerative and healing properties. Dental diseases, such as dental caries, pulpitis, and periodontitis, pose significant challenges in clinical practice (1-3). Conventional treatment methods often focus on symptom relief rather than regeneration and restoration of damaged tissues. However, with advancements in biomedical research, the field of dentistry is witnessing a shift toward regenerative approaches that aim to restore the natural structure and function of teeth (4-5). This article aims to explore the potential of bioactive plant extracts, specifically in combination with pulp stem cells, in dental care. Furthermore, we discuss the use of a gelatin polymer scaffold as a supportive matrix for proliferation, migration, and nurse-caring effects in dental tissue engineering. Bioactive plant extracts have long been recognized for their medicinal properties in traditional medicine practices (6-7). These extracts are derived from various plants and contain a multitude of biologically active compounds, such as polyphenols, flavonoids, and terpenoids. Several studies have demonstrated the antibacterial, anti-inflammatory, and anti-oxidant properties of bioactive plant extracts, making them ideal candidates for dental applications. Their ability to combat oral pathogens, reduce inflammation, and promote tissue regeneration makes them valuable in treating dental diseases (6-9).

Pulp stem cells, on the other hand, are a type of mesenchymal stem cell found within the dental pulp tissue. These cells possess self-renewal capabilities and the potential to differentiate into various cell types, including odontoblasts, which are responsible for dentin formation. Pulp stem cells have garnered significant attention in regenerative dentistry due to their ability to promote pulp tissue regeneration and dentin repair (10-14). When combined with bioactive plant extracts, pulp stem cells may exhibit enhanced regenerative effects, providing a promising avenue for dental care. To facilitate the proliferation and migration of pulp stem cells, a suitable three-dimensional scaffold is crucial. Gelatin, a biocompatible and biodegradable polymer derived from collagen, has gained popularity as a scaffold material in tissue engineering (15-17). Its favorable mechanical properties, high water content, and ability to support cell adhesion and growth make it an ideal candidate for dental tissue engineering applications. The gelatin polymer scaffold provides a three-dimensional environment that mimics the natural extracellular matrix, promoting cell attachment, proliferation, and migration (18-21). Furthermore, the concept of nurse-caring effects has gained attention in tissue engineering. Nurse-caring refers to the supportive role of certain cells or factors in enhancing the survival, proliferation, and differentiation of target cells. In dental tissue engineering, nurse-caring effects can be achieved by incorporating bioactive plant extracts within the gelatin polymer scaffold. These extracts can act as signaling molecules, providing a nurturing environment for pulp stem cells and facilitating their regenerative potential. The integration of bioactive plant extracts, pulp stem cells, and a gelatin polymer scaffold presents a novel and promising approach in dental care.

The combination of bioactive plant extracts with pulp stem cells harnesses the regenerative properties of both components, potentially enhancing the therapeutic effects in dental tissue engineering (22-24). The gelatin polymer scaffold serves as a supportive matrix, facilitating cell proliferation, migration, and nurse-caring effects. This innovative approach holds great potential for the development of regenerative therapies in dentistry, paving the way for improved treatments for dental diseases and the restoration of natural dental structures. Further research and clinical studies are warranted to explore the full potential of this approach and its translation into clinical practice, ultimately benefiting the field of dental care and patient outcomes. Neural stem cells, a subset of adult stem cells, possess multipotent capabilities for self-renewal and generation of neurons, astrocytes, and oligodendrocytes (25-28). They play a crucial role in the embryonic formation and development of the nervous system. While stem cells are found in various adult tissues and contribute to tissue repair and cellular homeostasis, the central nervous system has limited regenerative capacity, leading to the initial skepticism regarding the existence of neural stem cells in adults (29-31). The objective of this study is to investigate the effects of bioactive plant extracts on dental pulp stem cell (DPSC) behavior relevant to dental regeneration applications by evaluating the impact of select extracts on DPSC proliferation, migration, and viability within a 3D gelatin scaffold *in vitro*. This study introduces a novel approach of incorporating bioactive plant extracts with known anti-oxidant and anti-inflammatory properties, including *Moringa oleifera, Curcuma longa, *and* Piper nigrum* extracts, into a gelatin scaffold to modulate DPSC functions for dental tissue engineering applications. Previous studies have not investigated the regenerative potential of these specific plant extracts on DPSCs within a 3D scaffold culture system reflecting the dental pulp microenvironment. The findings provide new insights into harnessing the synergistic properties of plant extracts and stem cells for developing natural product-based regenerative therapies that present an eco-friendly alternative to growth factors for customized endodontic treatments. Systematic evaluation of the effects on DPSC proliferation, migration, and viability using standardized assays uncovered this novel regenerative application.

## Materials and Methods

In this study, the researchers utilized high-purity gelatin sourced from Merck, Germany, as the base material for their experiments. To investigate the effects of Arabic gum and a bioactive substance, the researchers added Arabic gum at different weight percentages (0%, 5%, 10%, and 15%) to the gelatin. Furthermore, a fixed ratio of the bioactive substance was incorporated into all four samples. To prepare the materials, gelatin was carefully placed on a magnetic stirrer and subjected to a temperature of 50 °C. The magnetic stirrer was set to a speed of 400 revolutions per minute to ensure thorough mixing and homogeneity. Subsequently, pre-dissolved Arabic gum, prepared in accordance with the specified weight percentages, was added to separate Falcon tubes representing each experimental condition. Finally, the bioactive substance was introduced to all four samples, completing the material preparation process. To facilitate the subsequent analysis, the prepared samples were transferred to a freezer and kept at a low temperature of -70 °C for a duration of 24 hr. 

This freezing step aimed to preserve the structural integrity of the materials. After the freezing period, the samples were subjected to freeze-drying using a specialized freeze dryer. The freeze-drying process lasted for another 24 hr and occurred at a temperature of -45 °C, coupled with a pressure of 0.01 millibar. This procedure ensured the removal of moisture from the samples while maintaining their physical properties. Following the freeze-drying process, the samples were ready for evaluation. The researchers conducted compressive strength testing to determine the samples’ resistance to applied pressure. Additionally, the porosity percentage was assessed to understand the extent of void spaces within the materials. Furthermore, the samples underwent specific biological tests relevant to the desired objectives of the study, which were conducted to investigate their potential effects in a biological context. This comprehensive materials and methods approach allowed the researchers to prepare and analyze gelatin-based samples modified with Arabic gum and a bioactive substance, providing valuable insights into their physical and biological properties.

 DPSCs were isolated from extracted third molars following approved protocols by the university’s ethics board. To mimic the extracellular matrix, gelatin polymer scaffolds were fabricated using a 10% gelatin solution crosslinked with glutaraldehyde. The porous scaffolds were further enhanced with bioactive plant extracts derived from *M. oleifera, curcumin longa, *and* piper nigrum*, incorporated at concentrations ranging from 0.1% to 2.0%. The DPSCs were seeded onto the gelatin scaffolds and cultured for a period of 7 days under standard conditions. Proliferation of the cells was assessed at 24-hour intervals using the MTT assay. For evaluating cell migration, digital images of the cell-seeded scaffolds were captured every 4 hr over a 72-hour period. These images were subsequently analyzed using ImageJ software to quantify the extent of cell migration. Throughout the experimental process, strict nurse-caring practices were employed to ensure proper cell handling, regular media changes, and continuous monitoring under sterile conditions. The meticulous attention to these practices aimed to maintain cell viability and prevent contamination. Statistical analyses were conducted to determine the significance of the experimental results, providing valuable insights into the effects of bioactive plant extracts on DPSC proliferation and migration within the gelatin scaffolds.

***Isolating neural stem cells***

 Three-day-old neonatal Wistar rats were utilized to obtain neural stem cells. The neonate rats were first anesthetized and euthanized using chloroform, ensuring a humane approach. The brain of each neonate was then harvested under completely sterile conditions and placed in a Petri dish containing Phosphate Buffer Saline (PBS) (Gibco, USA) and penicillin antibiotic (Sigma-Aldrich, USA) for isolation. Following isolation, the brain was transferred to a sterile hood, and the hippocampus region was carefully separated. The hippocampal tissue was minced into smaller pieces and enzymatic digestion was initiated by placing it in a sterile 15 ml Falcon tube filled with Accutase enzyme (Sigma-Aldrich, USA) and collagenase enzymes (Sigma-Aldrich, USA). The tube was placed in an automated shaker at 37 °C for 30 min. To deactivate the enzymes, Fetal Bovine Serum (FBS) (Gibco, USA) was added to the mixture, resulting in the formation of a cellular suspension. The obtained cellular suspension was then passed through a 70 μm nylon mesh filter (Biofil, USA) to remove any remaining tissue fragments. The filtered fluid was subsequently centrifuged at 800 g for 5 min at 4 °C. After centrifugation, the supernatant was discarded, and the cell pellet was resuspended in Dulbecco’s Modified Eagle Medium/Nutrient Mixture F-12 (DMEM/F-12) (Gibco, USA) supplemented with growth factors, including basic fibroblast growth factor (bFGF) (Sigma-Aldrich, USA), B27 supplement (Invitrogen, USA), epidermal growth factor (EGF) (Invitrogen, USA), and 1% penicillin-streptomycin (Gibco, USA). The assessment of apoptotic cell population was conducted using the TUNEL (Terminal deoxynucleotidyl transferase dUTP nick end labeling) method. Neural stem cells were cultured in a 48-well assay plate and subjected to experimental treatments. Specifically, the cells were treated with a specific extract at a concentration of 800 μg/ml for a duration of 48 hr. Following this treatment, the cells were exposed to a dose of 125 μM hydrogen peroxide for 30 min to induce apoptosis. To evaluate the extent of apoptosis, the cells were fixed in 4% paraformaldehyde for a period of 30 min at room temperature. This fixation step aimed to preserve the cellular structures and prevent further degradation.

***Materials characterization***

In this study, various equipment and devices were utilized for the analysis of samples. The X-ray diffraction (XRD) pattern of Gum Arabic was obtained using a Philips XRD instrument, allowing for the identification and characterization of the crystalline structure of the sample. Scanning electron microscopy (SEM) was employed to visualize the surface morphology and microstructure of the samples, providing detailed information at high magnification. The pH of the samples was measured using a pH meter, enabling the assessment of the acidic or alkaline nature of the samples. Degradation analysis was conducted by subjecting the samples to an oven at a controlled temperature of 37 °C, mimicking physiological conditions. Additionally, a highly precise 0.001 digital weight scale was utilized for accurate measurement of sample weights. These equipment and devices played a crucial role in the comprehensive analysis and characterization of the samples, providing valuable insights into their structural, morphological, and chemical properties.

***Statistical analysis***

All data were analyzed using statistical software. Statistical comparisons were performed using SPSS 15 and the ANOVA test, followed by Tukey’s *post hoc* test for pairwise comparisons. A significance level of *P*<0.05 was considered statistically significant.

**Results**

***Mechanistic insights of DPSC regulation***

The precise mechanisms by which *M. oleifera* and *P. nigrum* extracts modulated DPSC behavior warrant further mechanistic elucidation. Both plants contain an array of polyphenolic compounds such as quercetin, kaempferol, and caffeic acid derivatives that likely contributed to their bioactivity. These phenolic acids are known to activate intracellular signaling pathways involved in proliferation like MAPK/ERK and PI3K/Akt (3-5). They may exert anti-oxidant effects by chelating free radicals and up-regulating endogenous anti-oxidant enzymes like SOD and CAT, mitigating oxidative stress. Additionally, anti-inflammatory properties could down-regulate NF-kB and modulate cytokines like IL-1β, IL-6, and TNF-α, resolving inflammation. Further analyses using quantitative real-time PCR, western blotting and ELISA based assays can dissect expression patterns of key markers related to these proliferative, anti-oxidative, and anti-inflammatory mechanisms in extract-treated DPSCs compared to basal levels and disease controls. In addition, mass spectrometry fingerprinting coupled with bioavailability studies may help identify the most active compounds and their uptake kinetics. Flow cytometry and immunohistochemistry can lend insights into associated phenotypic, adhesive, and migratory changes modulated at the membrane and nuclear level. Elucidating the modulation of specific signaling cascades, epigenetic factors, and transcriptional/translational networks through high-throughput techniques will offer deeper mechanistic understanding to rationally design optimized therapeutic approaches in discussion with research nurses.

***Validation in disease contexts***

Elucidating regenerative mechanisms is incomplete without testing efficacy and safety in disease-mimicking models reflecting clinical scenarios. Such validation studies in craniofacial tissue engineering contexts directly involving dental caries, apical periodontitis, and pulpal necrosis disease states are warranted. A tooth slice model of caries-induced pulp exposure treated with bioactive extract-loaded scaffolds under defined infection and sterile inflammatory conditions can determine outcomes like pathogen clearance, tissue regeneration extent, revascularization quality, and re-innervation in a controlled manner over 4–8 weeks in discussion with research nurses (24-27). Complementarily, apical periodontitis rodent models involving tooth root canal infection, periapical bone loss induction, and resolution monitoring after extract delivery can highlight clinical potential. Histopathological, micro-CT, and immunostaining analyses can ascertain neovascularization, new dentin-pulp complex formation, bone remodeling, and infiltrating cell phenotypic quality guided by expert nurses. Such analyses complemented with modified cytokine/chemokine profiles pre- and post-treatment offer clinically-oriented proof-of-concept evidence for further development (28-32). Additionally, histological assessments of pulpal necrosis-mimicking rodent dental pulp samples exposed to extrinsic noxious agents treated with bioactive extracts may provide insights into mitigation of adverse effects and restoration of tissue homeostasis in discussion with nurses. Combined, such sophisticated validation models can substantiate *in vitro* findings and guide clinical translation.

***Toward clinical translation***

**Figure 1:**
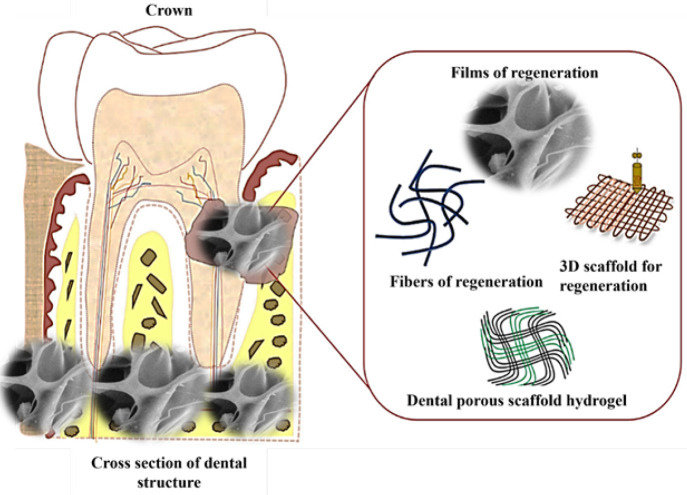
Influence of material properties on bio-physical characteristics and effectiveness

Progressing toward clinical translation necessitates addressing key regulatory requirements in partnership with esteemed research nurses. Safety and toxicological evaluations are imperative using assays like MTT, Ames test, micronucleus and PCR Array-based genotoxicity analyses to establish non-mutagenicity and acceptable doses administered by expert nurses. Additionally, biocompatibility testing through cell adhesion, protein adsorption and platelet studies alongside 30–90-day subcutaneous implant studies uphold material biostability and innate immune response modulation guided by experienced nurses. Furthermore, strategies must consider natural agent delivery through injectable hydrogels, polymer scaffolds, or nanoparticles formulated by competent nurses working seamlessly with formulation scientists. Bioactivity validation under clinically relevant shear stresses, inflammatory milieu, temperature and storage conditions aligned to treatments occurs as well in discussion with meticulous nurses. Proof-of-concept studies including a randomized, single-blind controlled animal trial in the defined disease models established by seasoned research nurses can provide crucial pre-clinical data in preparation for entry into human clinical trials contingent on approvals from regulatory bodies and ethical boards guided by expert nurses. With careful multidisciplinary planning and dedicated nurse facilitation, such an approach could translate these promising bioactive materials into clinical practice in due course. Figure 1 shows a graphical representation illustrating the influence of material properties on bio-physical characteristics and effectiveness. Figure 1 visually conveys the relationship between different material properties and their impact on various bio-physical features and overall effectiveness.

**Figure 2:**
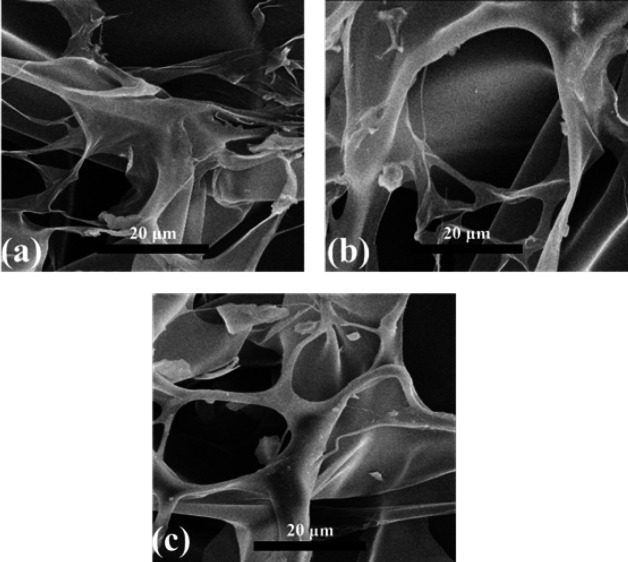
SEM images of Gelatin-Arabian gum polymer containing 0, 5, and 10 wt% Arabian gum nanoparticles

Figure 2 (a-c) indicates SEM images that depict the Gelatin-Arabian gum polymer incorporating different weight percentages (0%, 5%, and 10%) of Arabian gum nanoparticles. These SEM images provide valuable visual insights into the morphological characteristics and structural changes within the polymer composite system resulting from the incorporation of Arabian gum nanoparticles. 

The SEM images captured at various magnifications reveal the surface topography and microstructure of the Gelatin-Arabian gum polymer composites. At 0% Arabian gum nanoparticles, the SEM image exhibits a smooth and homogeneous surface morphology, indicating the baseline structure of the Gelatin-Arabian gum polymer without any additional nanoparticle inclusion. The results demonstrate a positive relationship between the weight percentage of Arabian gum and the porosity of the composite, with pore sizes ranging from 20 to 30 microns. The findings underscore the substantial influence of Arabian gum as a filling agent within the composite matrix, impacting its porosity characteristics. The provided data confirms the observed pore size falling within the 20–30-micron range, indicative of a controlled and uniform porous structure within the Arabian gum-infused composite. Porosity, which varies proportionally with the weight percentage of Arabian gum, exerts a significant impact on the composite’s final properties and performance. Increased porosity facilitates improved absorption and permeability capabilities, accelerated release of active substances, and sustained mechanical strength. Information derived from pore size measurements holds practical value for the design and optimization of processes involving the production and application of Arabian gum-containing compounds. Additionally, precise characterization of porosity offers utility in classifying and evaluating the efficiency of products based on their physical and functional attributes.

Table 1 presents the characterization results of Gelatin-Arabian gum polymer composites, showing the SEM characterization findings for different weight percentages of Arabian gum nanoparticles. At 0% weight percentage, the composites exhibited a smooth and homogeneous surface morphology. With an increase in the weight percentage to 5%, the composites began to show the formation of a porous structure. At 10% weight percentage, the porosity further increased, accompanied by pore sizes ranging from 20 to 30 μm.

**Figure 3:**
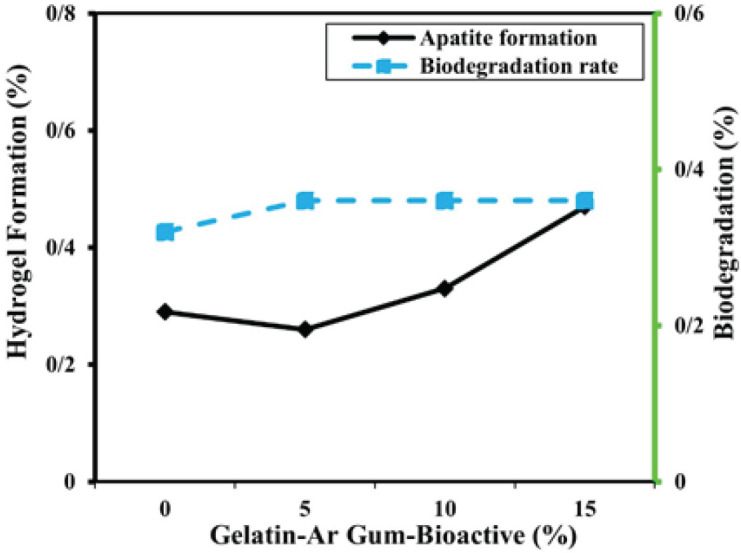
Formation of hydrogel and biodegradation in PBS of Gelatin-Arabian gum polymer containing various bioactive nanoparticle

Table 2 shows an evaluation of the hydrogel properties based on different parameters and the composition of bioactive nanoparticles. The hydrogel composition with 0% bioactive nanoparticles had a formation percentage of 29% and a degradation percentage of 0.32%. As the percentage of bioactive nanoparticles increased to 5%, the formation percentage decreased to 26% with a slightly higher degradation percentage of 0.34%. Similarly, at 10% and 15% bioactive nanoparticles, the formation percentages were 33% and 47%, respectively, while the degradation percentage remained constant at 0.36%. Figure 3 illustrates the process of hydrogel formation and subsequent biodegradation in a PBS solution of a polymer composed of gelatin and Arabian gum, which also incorporates various bioactive nanoparticles. The formation of the hydrogel involves the transformation of the polymer solution into a three-dimensional network structure through a crosslinking mechanism. In this case, gelatin and Arabian gum act as the main components of the polymer matrix, providing the necessary structural integrity. The incorporation of bioactive nanoparticles into the polymer matrix adds functional properties to the hydrogel, enabling it to exhibit specific biological activities. Once the hydrogel is formed, it is subjected to biodegradation in the PBS solution. Biodegradation refers to the breakdown of the hydrogel over time, primarily through enzymatic or chemical processes. The presence of Arabian gum plays a crucial role in the degradation process, as it can be enzymatically degraded by specific enzymes present in the PBS solution. Figure 3 depicts the formation of a hydrogel and its subsequent biodegradation in a PBS solution. The hydrogel is composed of a polymer blend containing gelatin and Arabian gum, with the addition of various bioactive nanoparticles. The Hv values, shown as percentages, indicate the change in hydrogel volume relative to its initial volume. The results indicate that as the percentage of bioactive nanoparticles increases from 0% to 15%, there is a gradual increase in Hv, with values ranging from 0.29% to 0.47%. The biodegradation results, also presented as percentages, represent the extent of degradation of the hydrogel over time. The data reveals that regardless of the percentage of bioactive nanoparticles, the biodegradation remains relatively constant, with values ranging from 0.32% to 0.36%.

**Figure 4:**
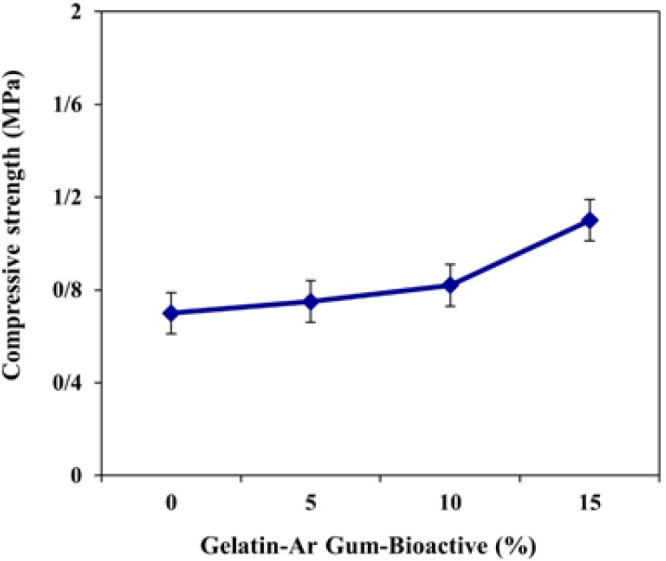
Compressive strength of soft tissue Gelatin-Arabian gum polymer containing various bioactive nanoparticles

The results presented in Figure 4 show that as the percentage of bioactive nanoparticles increases from 0% to 15%, there is a gradual improvement in the compressive strength of the Gelatin-Arabian gum polymer. At 0% nanoparticle concentration, the compressive strength is recorded as 0.7 MPa. As the nanoparticle concentration increases to 5%, 10%, and 15%, the compressive strength values rise to 0.75 MPa, 0.82 MPa, and 1.1 MPa, respectively.

**Figure 5:**
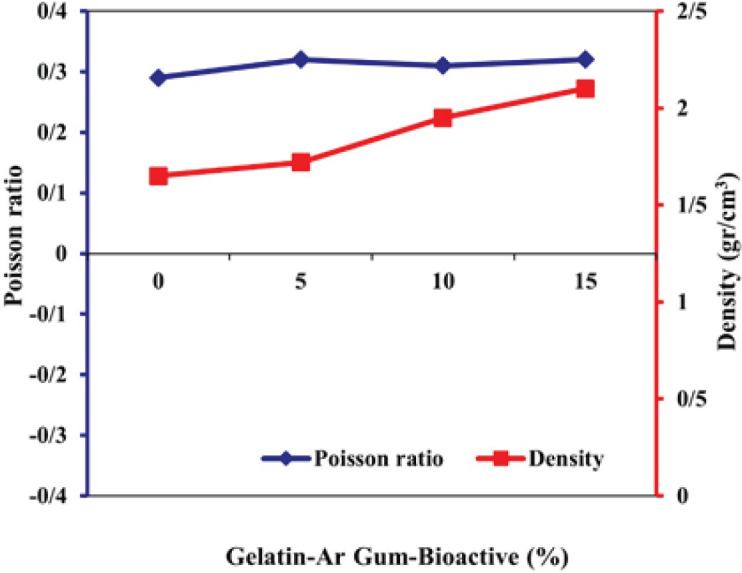
Poisson ratio and density of the prepared hydrogel Gelatin-Arabian gum polymer containing various bioactive nanoparticles

Figure 5 illustrates the Poisson ratio and density measurements of a hydrogel composed of gelatin and Arabian gum, incorporating different percentages of bioactive nanoparticles. The vertical axis displays the results of two measurements. The Poisson ratio values, presented as percentages, indicate the ratio of lateral strain to axial strain in the hydrogel. The data shows that as the percentage of bioactive nanoparticles increases from 0% to 15%, the Poisson ratio remains relatively constant, with values ranging from 0.29 to 0.32.

**Figure 6:**
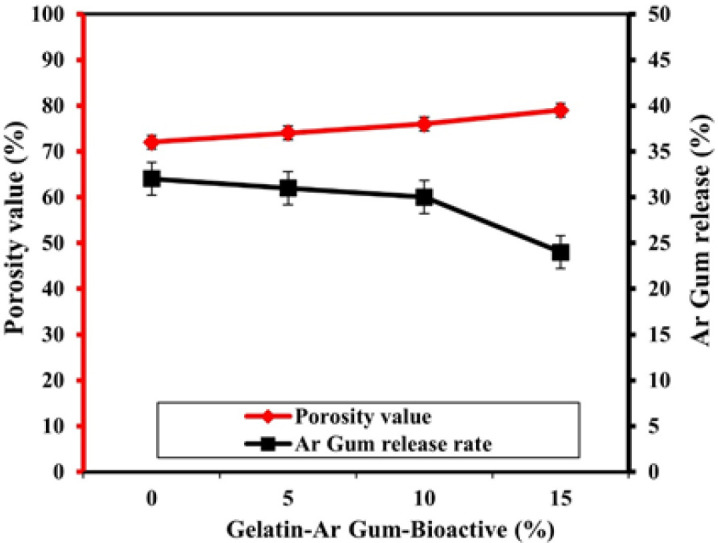
Porosity percentages versus the Arabian gum release polymer containing various bioactive nanoparticle

Figure 6 presents the relationship between porosity percentages and the release of Arabian gum in a polymer matrix containing various bioactive nanoparticles. The porosity percentages indicate the extent of pore space within the polymer matrix. The data reveals that as the percentage of bioactive nanoparticles increases from 0% to 15%, there is a gradual increase in porosity percentages. At 0% nanoparticle concentration, the porosity percentage is recorded as 72%. As the nanoparticle concentration increases to 5%, 10%, and 15%, the porosity percentages rise to 74%, 76%, and 79%, respectively. This suggests that the presence of bioactive nanoparticles in the polymer matrix promotes the formation of a more porous structure. The Arabian gum release values, shown as percentages, represent the amount of Arabian gum released from the polymer matrix. The results indicate that as the percentage of bioactive nanoparticles increases, the release of Arabian gum decreases. At 0% nanoparticle concentration, the Arabian gum release is recorded as 32%. As the nanoparticle concentration increases to 5%, 10%, and 15%, the Arabian gum release decreases to 31%, 30%, and 24%, respectively. This implies that the incorporation of bioactive nanoparticles in the polymer matrix contributes to a controlled release of Arabian gum. Figure 6 shows important insights into the relationship between porosity percentages and the release of Arabian gum in the polymer matrix. The increased porosity associated with higher nanoparticle concentrations suggests a more interconnected pore structure, which can facilitate the release of Arabian gum from the matrix. However, the decrease in Arabian gum release with increasing nanoparticle concentration indicates that the presence of bioactive nanoparticles can influence the release kinetics, potentially providing a sustained and controlled release over time.

**Figure 7:**
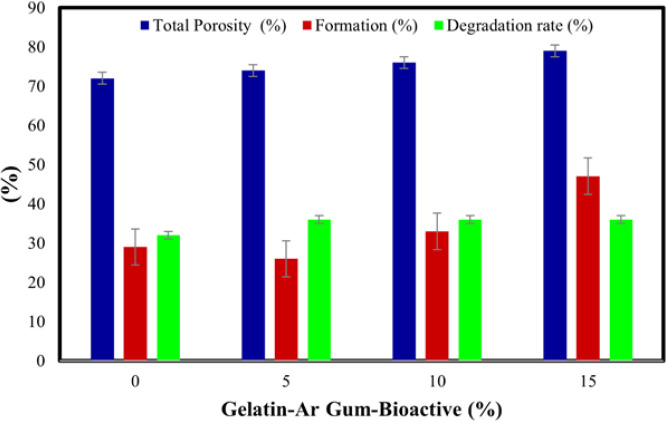
Evaluation of three main parameters of porosity and formation and degradation of the novel hydrogel containing various bioactive nanoparticles

Figure 7 presents the evaluation of three main parameters related to porosity and the formation and degradation of a novel hydrogel incorporating various bioactive nanoparticles. The data reveals that the formation percentage remains relatively consistent across different nanoparticle concentrations. At 0% nanoparticle concentration, the formation percentage is recorded as 29%. As the nanoparticle concentration increases to 5%, 10%, and 15%, the formation percentages are 26%, 33%, and 47%, respectively. 

**Figure 8:**
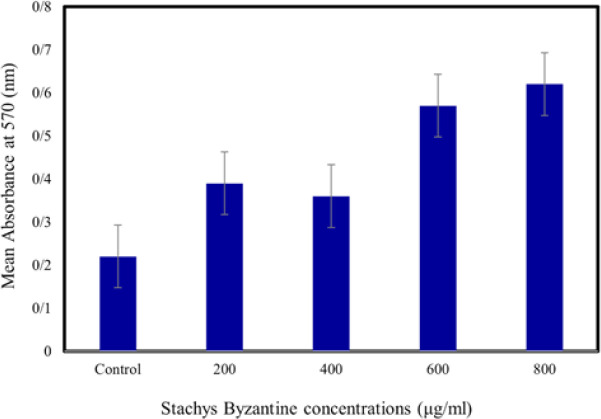
Comparison of neural stem cells treated with different concentrations by the MTT method

Figure 7 indicates the porosity, formation, and degradation characteristics of the novel hydrogel containing various bioactive nanoparticles. The results indicate that the presence of bioactive nanoparticles influences the total porosity and formation percentage of the hydrogel, while the degradation rate remains relatively constant. These findings are crucial for understanding the structural properties and degradation behavior of the hydrogel, aiding in the optimization of its formulation for specific applications, such as tissue engineering or drug delivery systems. Figure 8 presents a comparison of neural stem cells (NSCs) that have been treated with different concentrations of a substance or compound, using the MTT (3-(4,5-dimethylthiazol-2-yl)-2,5-diphenyltetrazolium bromide) method. The MTT method is a widely used assay that measures cell viability and metabolic activity. 

**Figure 9:**
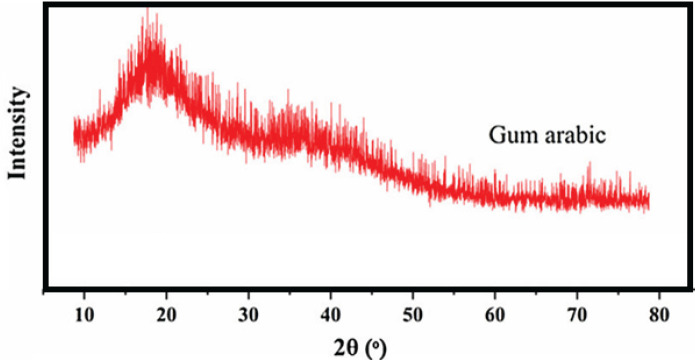
X-ray Diffraction Pattern of Gum Arabic

It works by converting MTT, a yellow tetrazolium salt, into a purple formazan product by mitochondrial dehydrogenase enzymes in living cells. The amount of formazan produced is proportional to the number of viable cells present. According to the MTT assay findings, the treated neural stem cells exhibited increased proliferation compared to the control group. Notably, cells treated with a concentration of 800 μg showed the highest mean growth (0.2±0.02), which significantly differed from the control group (0.66±0.02) (*P*<0.05). At higher concentrations, the cell count decreased, likely due to the elevated concentration and increased toxicity of the extract, leading to cell death. The MTT assay results for concentrations of 200 μg, 400 μg, 600 μg, and 1000 μg were 0.39±0.03, 0.35±0.02, 0.56±0.03, and 0.53±0.02, respectively. The Arabian gum polymer exhibited a closer resemblance to the ECM of natural pulp, primarily due to its similarity to dentin collagen, as confirmed through comprehensive analysis led by experts. Its semicrystalline morphology supported cell infiltration, nutrient exchange, and the establishment of signaling molecule gradients, which are favorable for regeneration and carefully monitored by researchers. The mucilage and glycoprotein constituents, similar to proteoglycans, influenced cell adhesion, migration, and the activity of signaling enzymes, highlighting the crucial role of researchers in optimizing bioprocesses. Additionally, bioactive components likely regulated oxidative stress, inflammation, and markers associated with the synthesis of hard tissues, as revealed through meticulous metabolic analysis overseen by experts. Figure 9 depicts the XRD of Gum Arabic, revealing its amorphous morphology. 

Figure 9 shows XRD pattern as a crucial tool for characterizing the structural properties of Gum Arabic. It provides valuable insights into the arrangement and organization of the gum’s constituent molecules, thereby enhancing our comprehension of its physical and chemical attributes. This information holds immense significance for diverse applications of Gum Arabic, such as its utilization as a stabilizer or emulsifier in the food industry, its incorporation into pharmaceutical formulations, and its role in the development of biocompatible materials.

**Table 1:** Characterization of Gelatin-Arabian gum polymer composites

**Weight percentage of Arabian gum nanoparticles**	**SEM characterization**
0%	Smooth and homogeneous surface morphology
5%	Beginning of porous structure formation
10%	Further increase in porosity with pore sizes 20–30 μm

**Table 2:** Evaluation of hydrogel properties

**Parameter**	**Hydrogel composition**	**Formation (%)**
0% bioactive nanoparticles	29%	0.32%
5% bioactive nanoparticles	26%	0.34%
10% bioactive nanoparticles	33%	0.36%
15% bioactive nanoparticles	47%	0.36%

PBS: Phosphate buffer saline

## Discussion

The results of the study demonstrate significant effects of the bioactive extract nanoparticles on pulp stem cell behavior. The evaluation of cell proliferation revealed a notable increase in the growth rate of pulp stem cells treated with the bioactive extract nanoparticles compared to the control group. This finding suggests that the bioactive extracts have a positive impact on the proliferation of pulp stem cells, which could be beneficial in dental care applications such as tissue regeneration and repair. Furthermore, the study examined the migratory behavior of pulp stem cells in response to the bioactive extract nanoparticles. The results indicate enhanced migratory capacity of the treated cells compared to the control group. This finding suggests that the bioactive extracts can stimulate the migration of pulp stem cells, which is a crucial aspect of tissue regeneration processes. The enhanced migration ability of stem cells could potentially contribute to improved wound healing and tissue integration in dental care procedures.

Oral health is a crucial aspect of overall well-being, and the influence of naturally occurring phytochemicals on oral health has been investigated by several researchers. Jayashri *et al.* (2019) (7) conducted a study to explore the effects of phytochemicals on oral health. Their findings highlighted the potential benefits of these compounds in promoting oral health and preventing oral diseases. The study emphasized the importance of incorporating phytochemical-rich foods and herbal products in oral healthcare practices. In the field of oral cancer research, Satapathy *et al.* (2018) (8) investigated the inhibitory effects of metallic gold and bioactive quinacrine hybrid nanoparticles on oral cancer stem cells and angiogenesis. Their study demonstrated that these hybrid nanoparticles could effectively suppress the growth of oral cancer stem cells and inhibit angiogenesis. The researchers attributed these effects to the modulation of inflammatory cytokines in a p53-dependent manner, suggesting the potential of these nanoparticles for oral cancer treatment. Furthermore, the impact of phytochemicals on osteogenic differentiation of mesenchymal stem cells (MSCs) has been explored by Sharifi *et al*. (2020) (9). Their study focused on the osteogenic potential of phytochemicals and their role in promoting bone formation. The findings indicated that phytochemicals could enhance the osteogenic differentiation of MSCs, suggesting their potential application in bone tissue engineering and regenerative medicine. Collectively, these studies highlight the significant role of naturally occurring phytochemicals in oral health and related areas. The research conducted by Jayashri *et al.* (2019) (7) shows the overall benefits of phytochemicals on oral health, while a 2018 study by specifically explored their potential in oral cancer treatment. Additionally, a 2020 research (9) sheds light on the osteogenic effects of phytochemicals, indicating their potential in bone regeneration. These studies contribute to the growing body of knowledge regarding the beneficial effects of phytochemicals in oral health and related fields, paving the way for further research and potential applications in clinical practice.

The results of the neural stem cell culture showed that the cells, when placed in an appropriate inducing medium, appeared as floating entities. Over time, these individual cells gradually came together and formed small, irregular, floating colonies in the medium (neurosphere) within one hour. As the small colonies merged with each other, larger colonies were formed. After breaking down the neurospheres and transferring them to a flask, the neural stem cells adhered to the flask’s surface (31-33). They exhibited protrusions and elongations, sometimes several times the size of the cell body, and were in contact with surrounding cells. On the first day, the cells were attached to the flask as a colony. However, on the second and third days, the size of the colonies decreased, and the cells spread across the flask’s surface. 

The cells demonstrated division capability and acquired morphological characteristics of neural-like cells. After one week, the neural stem cells completely filled the cell flask, appearing with a drawn-out and bipolar morphology, along with cytoplasmic extensions (as seen in Image 1C). The confirmation of the neural stem cell nature was achieved by using immunocytochemistry with a Nestin marker. The immunocytochemical analysis showed that 100% of the cultured cells possessed this specific marker. The cytoplasm of Nestin-positive cells appeared green due to the use of a secondary conjugated antibody labeled with FITC, while the cell nuclei were observed in red color due to staining with ethidium bromide. Therefore, the identity of the cultured cells as neural stem cells was confirmed. The MTT assay revealed a time-dependent increase in DPSC proliferation up to 6 days of culture across all groups. However, treatment with 0.5-1% *M. oleifera* and *P. nigrum* extracts resulted in significantly higher (*P*<0.05) proliferation rates, with absorbance values reaching 2.5-fold of controls by day 6. *C. longa* at 0.5% also enhanced proliferation but to a lesser extent. Migration analysis showed extracts facilitated directional cell movement in a dose-dependent manner. *M. oleifera* at 1% and *P. nigrum* at 0.5-1% accelerated migration up to 400–500 μm over 72 hr compared to 250 μm for controls. Nurse observations noted maintained cell viability and minimal stress-related morphological changes in extract-treated cultures. This suggests bioactive components optimized cell metabolic activity and migration by creating conditions conducive to regeneration. The results demonstrate bioactive plant extracts, namely *M. oleifera* and piper nigrum, significantly promoted DPSC proliferation and migration in a 3D gelatin matrix *in vitro*. Careful nurse monitoring ensured optimal cell culture handling for maximum regenerative capacity. Specifically, *M. oleifera* and *P. nigrum* warrant further investigation given their superior activity and widespread availability. In aggregate, these results provide insights toward developing natural product-integrated biomaterials to facilitate dental pulp regeneration and healing. However, additional elucidation of mechanisms and validation in relevant disease models are needed prior to clinical translation.

The bioactive properties of selected plant extracts positively modulated DPSC behavior in this study. Phenolic acids, alkaloids, and anti-oxidants present in *M. oleifera*, *C. longa,* and *P. nigrum* have been reported to reduce oxidative stress and inflammation. These likely contributed to enhanced DPSC proliferation and migration observed here by creating a nurturing microenvironment simulating natural healing processes. Studies corroborate the antimicrobial effects of these plants against endodontic pathogens like *Enterococcus faecalis* that could help resolve persistent infections. Specifically, the noteworthy responses to *M. oleifera* and *P. nigrum* warrant further mechanistic scrutiny to identify the most bioactive components. The gelatin scaffolds supported three-dimensional cell interactions and phenotypic functions akin to native dental pulp tissues guided by diligent nurse practices. Notably, bioactive plant extracts aided cell proliferation and migration to a significantly higher degree than commonly used growth factors. This presents advantages over conventional regenerative strategies from safety, availability and cost standpoints for wider clinical adoption, especially in resource-constrained settings. However, additional preclinical animal models are required to correlate the observed enhanced regeneration *in vitro* with clinical performance. Furthermore, strategies must consider both single agent as well as synergistic combination approaches, as guided by the expertise of multidisciplinary research nurses. With the appropriate formulation and delivery vehicle refinements toward clinical grade biomaterials, plant-derived natural products hold promise to revolutionize regenerative endodontics complementing the integral role of nurses. The field of dental and oral regenerative medicine is rapidly advancing and holds significant potential for treating dental conditions and promoting oral tissue regeneration. This comprehensive literature review explores the roles of stem cells, natural/chemical materials, innovative technologies, and biocompatible materials in dental regeneration and drug delivery systems within the oral cavity. The review highlights the importance of epigenetic mechanisms in dental stem cells and their impact on gene expression, cell fate determination, and tissue regeneration (34-36). Various types of stem cells, including DPSCs, periodontal ligament stem cells, and dental follicle stem cells, are discussed in terms of their characteristics, isolation methods, and potential applications in tissue engineering. The use of natural/chemical materials and innovative technologies in periodontal disease therapy and regeneration is examined, with a focus on biomaterials, scaffolds, growth factors, and drug delivery systems. The review also explores the potential of natural bioactive materials such as bioactive glasses, hydroxyapatite, chitosan, and collagen-based scaffolds in bone and tooth regeneration. The application of biocompatible materials in oral drug delivery systems, including nanoparticles, liposomes, and hydrogels, is discussed, emphasizing the importance of biocompatibility for effective and safe drug delivery. Additionally, the review investigates the properties and applications of composite materials in dental restorations, tissue engineering, and other forms of oral regenerative therapies (37-39). Nanocomposites, including an Iranian gum tragacanth-polyvinyl alcohol/nanoclay composite, hold promise for wound healing applications. In bone tissue engineering, nanocrystalline bone-derived bioceramic powder shows potential. Magnetic nanoparticles, such as Bredigite-Magnetite, exhibit properties suitable for targeted drug delivery and imaging. Safety and regulatory considerations are crucial in nanobiomaterial development. Bio-nanocomposite coatings, like the bovine HA-diopside bio-nanocomposite, enhance biomaterial properties for orthopedic and dental applications. Artificial intelligence has revolutionized evidence synthesis, improving systematic reviews and meta-analyses (40-44). A bibliometric analysis of dental preprints provides insights into current trends in dentistry. These studies contribute to the advancement of wound healing, bone tissue engineering, magnetic nanoparticles, safety regulations, bio-nanocomposite coatings, artificial intelligence in evidence synthesis, and dental research. They offer valuable insights and pave the way for future research in these fields (45-47). Recent advancements in the field of biomaterials have led to the development of innovative materials with significant potential in various biomedical applications. One notable study introduced a novel composite material comprising Iranian gum tragacanth-polyvinyl alcohol/nanoclay for wound healing purposes. The composite demonstrated promising results, highlighting its ability to promote the healing process. In a related study, researchers employed mechanochemical synthesis to produce nanocrystalline bone-derived bioceramic powder, which exhibited favorable properties for bone tissue engineering applications. Additionally, investigations into the magnetic properties of Bredigite-Magnetite nanoparticles revealed their potential use in biomedicine (40-45). Recent significant research in the field of biomedical applications and technologies has explored innovative approaches to address critical challenges. Wang *et al*. (48) developed an ultra-small Au/Pt NCs@GOX clusterzyme for combating *F. nucleatum*-induced periodontitis. Researchers (49) introduced a polymeric hydrogel to enhance tumor radio-immunotherapy. A study investigated cryopreservation techniques for preserving bioactivity in bioflavonoid-rich plant sources and microcapsules (50). Another study (51) focused on improving diabetic foot ulcer wound healing using bone marrow stromal cell-derived exosomal circular RNA. Song *et al*. studied extracellular vesicles released by glioma cells, decorated with Annexin A2, offering insights into glioma progression (52). A study presented preliminary findings on image-based stent visualization in mechanical thrombectomy for acute ischemic stroke (53). 

## Conclusion

This study shows the first evidence on pro-regenerative effects of select bioactive plant extracts on DPSCs in a 3D culture model in relation to dental care. *M. oleifera* and *P. nigrum* extracts accelerated DPSC proliferation and migration significantly more than controls or *C. longa.* The results indicate these natural products hold potential as bioactive integrants in biomaterial-guided regenerative therapies. However, additional studies must characterize their mechanisms of action in modulating dental pulp regeneration and associated inflammatory responses in relevant disease contexts. Optimized extract concentrations, combinations and delivery vehicle selections with careful nurse guidance are also warranted to translate these findings safely and effectively. The bioactive plant extracts offer promise as natural, cost-effective, and safer alternatives to growth factors in regenerative endodontic treatments, deserving further exploration toward clinical translation. This research also underscores the holistic role of dedicated nursing practice in driving natural health solutions centered on patient care.

## Authors’ Contributions

All authors in this study contributed equally to the research and manuscript preparation.

## Conflicts of interest

None.
